# Effects of whole cycle nutrition management based on “Hospital to Home (H2H)” model on nutritional status and immune function of patients with gastrointestinal tumor chemotherapy

**DOI:** 10.3389/fmed.2026.1775700

**Published:** 2026-03-31

**Authors:** Shan Wang, Juan Cheng, Yu Guo, Wenrui Suo

**Affiliations:** Department of General Surgery and Gastroenterology, Suqian Hospital of Jiangsu Provincial People’s Hospital, Suqian, China

**Keywords:** chemotherapy, colorectal cancer, gastrointestinal tumor, gastric cancer, hospital to home, immune function, nutrition management

## Abstract

**Aim:**

To evaluate effects of whole-cycle nutrition management based on “Hospital to Home (H2H)” model on nutritional status and immune function in gastric and colorectal cancer patients receiving chemotherapy.

**Methods:**

In this randomized controlled trial, 100 gastric or colorectal cancer patients with malnutrition risk (PG-SGA ≥ 2) were randomly assigned to routine nursing (control, *n* = 50) or H2H nutrition management plus routine nursing (study, *n* = 50). Primary outcomes were PG-SGA score and serum albumin. Secondary outcomes included immune function (CD4+, CD8 + counts, CD4+/CD8 + ratio), hematological parameters, cancer-related fatigue (PFS), self-care ability (ESCA), quality of life (GQOLI-74), adverse reactions (CTCAE 5.0), and nursing satisfaction. Linear mixed-effects models with FDR correction were used for repeated measures.

**Results:**

The study group showed significantly greater improvements in PG-SGA score (mean difference at cycle 6: −1.4, 95% CI: −1.9 to −0.9, *p* < 0.001) and serum albumin (+2.7 g/L, 95% CI: 1.5 to 3.9, *p* < 0.001). CD4 + counts (+95 cells/μL, *p* < 0.001) and CD4+/CD8 + ratio (+0.35, *p* < 0.001) were significantly higher in the study group. Cancer-related fatigue (PFS) decreased, while self-care ability (ESCA) and quality of life (GQOLI-74) improved more in the study group (all q < 0.05). Adverse reactions were lower (4.0% vs. 20.0%, *p* = 0.01) and nursing satisfaction higher (96.0% vs. 80.0%, *p* = 0.01) in the study group.

**Conclusion:**

H2H whole-cycle nutrition management improves nutritional status, immune function, fatigue, self-care ability, quality of life, and satisfaction while reducing adverse reactions in gastric and colorectal cancer patients receiving chemotherapy. Larger multicenter studies with long-term follow-up are needed to confirm these findings and assess survival outcomes.

## Introduction

According to the survey data of the “2020 Global Cancer Statistics Report”, cancer is becoming a kind of the main causes of death all over the world, gastrointestinal cancer has become the fifth largest cancer in the world (1), and the situation of gastrointestinal cancer in China is more severe, with the number of new cases reaching 479,000 in 2020 and the number of deaths reaching 374,000, with the incidence and mortality ranking third among malignant tumors (2). Due to the dual effects of tumor and chemotherapy, about 31 to 87% of patients with malignant tumors have different degrees of malnutrition during adjuvant chemotherapy, and the incidence of malnutrition in digestive tract tumors is the first (3). Malnutrition will not only reduce body function, but also affect the metabolism of chemotherapy drugs, diminish their tolerance, aggravate adverse reactions, as well as adversely affect patients’ prognosis and quality of life (4). Because of the long treatment time of chemotherapy patients, home nutrition management is very important, which has a positive role in promoting physiological function and optimizing health resources (5). In recent years, nutrition management has become an indispensable part of the comprehensive treatment of cancer (6).

With the continuous development of medicine, nutritional therapy has become a first-line clinical treatment (7). Studies have shown that nutritional management is the most economical and effective measure to prevent disease complications and delay tumor progression in clinical practice, and is also the most acceptable management method for patients (8). However, because chemotherapy patients have a long treatment interval between two courses, patients spend more time recuperating at home than in hospital, and nursing staff have less intervention, which makes it difficult for patients to continue nutrition education and nutrition support for a long time, resulting in the interruption of nutrition management of patients during the interval of chemotherapy, resulting in scattered and unsystematic nutrition management (9). Therefore, nutritional care of patients with gastrointestinal tumor chemotherapy needs continuous personalized guidance.

At present, nutritional management for patients with gastrointestinal tumors mainly focuses on the perioperative period or chemotherapy period and mostly adopts streamlined nutritional management (10). However, continuous nursing after discharge is a weak link, lacking nutrition attention during the home period, and lacking continuous nutrition management in hospital and outside hospital (11). The Hospital to Home (H2H) nutrition management model was first proposed by the Nutrition Department of West China Hospital (12). It is a continuous and personalized nutrition management model from hospital to family, which extends the nutritional treatment of patients from hospital to family, and has been preliminarily utilized in patients with chronic diseases (13).

In this study, the “H2H” nutritional management mode was applied to patients with gastrointestinal tumor chemotherapy to study the impacts of this mode on improving the nutritional status and quality of life of patients and provide a new basis for future nutritional management.

## Data and methods

### Study design and registration

This study was designed as a single-center, randomized, two-arm parallel-group controlled trial. The study protocol was approved by the Ethics Committee of the Suqian Hospital of Jiangsu Provincial People’s Hospital (Approval No. 2025-SR-0345) and was conducted in accordance with the Declaration of Helsinki. All participants provided written informed consent before enrollment. The study was registered with the Chinese Clinical Trial Registry. The reporting of this trial follows the CONSORT (Consolidated Standards of Reporting Trials) guidelines.

### Participant flow

A total of 158 patients with gastrointestinal tumors admitted to our department from March 1, 2024 to April 30, 2025 were assessed for eligibility. Of these, 58 patients were excluded: 32 did not meet the inclusion criteria, 20 declined to participate, and 6 were excluded for other reasons (e.g., transfer to other hospitals, scheduling conflicts). The remaining 100 eligible patients were randomly assigned to the control group (*n* = 50) or the study group (*n* = 50). All 100 patients received their allocated interventions. During follow-up, 1 patient in the control group and 2 patients in the study group were lost to follow-up due to withdrawal of consent. All 100 patients were included in the final analysis (intention-to-treat principle), as the linear mixed-effects model handles missing data under the missing-at-random assumption. The participant flow diagram is presented in Supplementary Figure S1.

### Inclusion and exclusion criteria

*Inclusion criteria*: (1) Age 18–75 years; (2) histopathologically confirmed diagnosis of gastric adenocarcinoma or colorectal adenocarcinoma (including colon and rectal cancer); (3) receiving their first perioperative chemotherapy, specifically: (a) neoadjuvant chemotherapy for resectable gastric or colorectal cancer, administered by the Enhanced Recovery After Surgery (ERAS) multidisciplinary team; or (b) adjuvant chemotherapy following radical resection of gastric or colorectal cancer, initiated within 4–8 weeks after surgery; (4) volunteered to participate in this study and provided written informed consent; (5) clear consciousness, good mental status, and no communication barriers; (6) Patient-Generated Subjective Global Assessment (PG-SGA) ≥ 2 scores (14), indicating at least mild malnutrition risk.

*Exclusion criteria*: (1) Presence of other malignant tumors (excluding non-melanoma skin cancer or carcinoma *in situ* of the cervix), diabetes mellitus, gout, or acquired immune deficiency syndrome (AIDS); (2) history of organ transplantation; (3) diagnosis of depression, anxiety disorders, or cognitive impairment that would interfere with study participation; (4) perioperative death; (5) incomplete clinical data (missing >20% of planned assessments); (6) poor compliance (failure to attend scheduled chemotherapy sessions or follow nutrition recommendations on ≥2 occasions); (7) received preoperative radiotherapy or concurrent chemoradiotherapy; (8) participated in other interventional clinical trials during the study period.

### Randomization and allocation concealment

After providing written informed consent and completing baseline assessments, eligible patients were randomly assigned to either the control group or the study group in a 1:1 ratio. The randomization sequence was generated using a computer-generated random number table (SPSS version 26.0, IBM Corp., Armonk, NY, USA). To minimize potential confounding, we used stratified block randomization with a block size of four, stratifying by tumor site (gastric cancer vs. colorectal cancer vs. others) and baseline nutritional risk (PG-SGA score 2–3 vs. ≥4). The allocation sequence was prepared by an independent statistician who was not involved in patient recruitment, intervention implementation, or outcome assessment.

Allocation concealment was ensured using sequentially numbered, opaque, sealed envelopes (SNOSE). Each envelope contained the group assignment and was labeled with a unique study ID number. After a patient met all eligibility criteria and provided written informed consent, a research nurse who was responsible for enrollment opened the next envelope in sequence in the presence of the patient to reveal group allocation. This process ensured that the individuals enrolling participants were unaware of the upcoming assignment, thereby minimizing selection bias.

### Chemotherapy regimens and supportive care

All patients received standardized chemotherapy regimens according to the National Comprehensive Cancer Network (NCCN) guidelines for gastric or colorectal cancer. The specific regimens were as follows. (1) For gastric cancer patients: SOX regimen: S-1 (40–60 mg twice daily, days 1–14) + oxaliplatin (130 mg/m^2^, day 1), repeated every 3 weeks. XELOX regimen: capecitabine (1,000 mg/m^2^ twice daily, days 1–14) + oxaliplatin (130 mg/m^2^, day 1), repeated every 3 weeks. (2) For colorectal cancer patients: FOLFOX6 regimen: oxaliplatin (85 mg/m^2^, day 1) + leucovorin (400 mg/m^2^, day 1) + 5-FU (400 mg/m^2^ bolus, then 2,400 mg/m^2^ continuous infusion over 46 h), repeated every 2 weeks. XELOX regimen: capecitabine (1,000 mg/m^2^ twice daily, days 1–14) + oxaliplatin (130 mg/m^2^, day 1), repeated every 3 weeks. FOLFIRI regimen: irinotecan (180 mg/m^2^, day 1) + leucovorin (400 mg/m^2^, day 1) + 5-FU (400 mg/m^2^ bolus, then 2,400 mg/m^2^ continuous infusion over 46 h), repeated every 2 weeks.

All patients received standard supportive care, including: Antiemetic prophylaxis: 5-HT3 receptor antagonist (ondansetron 8 mg or palonosetron 0.25 mg) + dexamethasone (5–10 mg) before each chemotherapy cycle. Granulocyte colony-stimulating factor (G-CSF): administered prophylactically if neutropenia occurred in previous cycles or therapeutically for grade ≥3 neutropenia, according to institutional protocols. Erythropoiesis-stimulating agents (ESA): used only for patients with hemoglobin <8 g/dL or symptomatic anemia, at the discretion of treating physicians. Blood transfusions: administered for hemoglobin <7 g/dL or as clinically indicated.

The use of supportive medications (G-CSF, ESA, transfusions) was systematically recorded for all patients throughout the study period.

### Intervention methods

The control group adopted routine nursing, and routine nutrition management was adopted, including nutrition risk screening, diet guidance and nutrition education.

The study group adopted “H2H” nutritional management mode plus routine nursing, the specific methods were as follows:

1. A multidisciplinary nutrition management team was set up in the hospital, and PG-SGA assessment was performed before each chemotherapy. The corresponding nutritional support program, namely five-step therapy, was determined according to the score. A score of 0–1 meant without nutritional intervention. A score of 2–3 meant suspected malnutrition, which was the first step: patients and their families were required to receive nutrition education. A score of 4–8 indicated moderate malnutrition. At this time, it was calculated whether the actual energy intake of the patient met the target requirement, and enteral nutrition (EN) and parenteral nutrition (PN) were performed on the patient. Patients and family members were taught to keep food diaries.
2. A chemotherapy WeChat group was established, and relevant nutrition knowledge were pushed regularly. Patients were guided to keep a food diary after returning home from hospital. When the current ladder failed to reach 60% of the target energy demand for 3–5 days, patients were guided to enter the second ladder: diet + oral nutritional supplement (ONS), the dose was at least 400–600 kcal/d, and patients were recommended to take it between three meals. If the current steps still could not meet the patient’s needs, patients were informed to return to the hospital for evaluation by the medical team.
3. According to the patients’ eating habits and dietary preferences, combined with the nutritional assessment of the patients by the nutritional team and the nutritional indexes (body mass index (BMI), serum albumin, hemoglobin) in the hospital, the patients’ nutritional status was assessed, and the diet plan was formulated. Patients were instructed to prepare their own body mass scale, record fasting body mass every morning, guide the record and upload it every week, which were collected by a special person, and the team members made dynamic adjustment and timely feedback according to the nutritional status and intake of patients. Patients in urban areas were followed up at home, and patients around urban areas and other places were mainly followed by phone + WeChat video.
4. Physical analysis was performed continuously before the first, third and sixth courses of treatment. Weight, BMI, blood biochemical indexes and other values were collected each time patients came to the hospital for chemotherapy. For patients with continuous body mass decline, nutritional support was assessed and analyzed by the nutrition team.

### Sample size calculation

The sample size was calculated based on the primary outcome of PG-SGA score change. According to our preliminary pilot study (*n* = 20 per group), the mean PG-SGA score after intervention in the control group was 5.2 with a standard deviation (SD) of 1.8. We considered a clinically meaningful reduction of 1.2 points (approximately 0.67 SD) in the study group compared to the control group to be relevant, based on previous literature on nutrition interventions in cancer patients.

Using a two-sample *t*-test with a two-sided significance level (*α*) of 0.05 and power (1-*β*) of 0.80, the required sample size per group was calculated as:


$$\begin{array}{l} n=2\times {\left(Z\_(1-\alpha /2)+Z\_(1-\beta )\right)}^{2}\times {\left(\sigma /\delta \right)}^{2}\\ =2\times {\left(1.96+0.84\right)}^{2}\times {\left(1.8/1.2\right)}^{2}=40\;\mathrm{per}\;\text{group}. \end{array}$$

where Z_(1-α/2) = 1.96 for α = 0.05, Z_(1-β) = 0.84 for β = 0.20, σ = 1.8 (pooled SD), and δ = 1.2 (minimum clinically important difference).

Accounting for an anticipated dropout rate of 20% during the 6-month follow-up period, we inflated the sample size by 25%:


Final sample size=40/(1–0.20)=50pergroup.

Therefore, a total of 100 patients (50 per group) were required for this study. Sample size calculation was performed using G*Power software (version 3.1.9.7, Heinrich-Heine-Universität Düsseldorf, Germany).

### Study outcomes and measurements

The outcomes of this study were classified as primary and secondary endpoints to control for type I error inflation. All measurements were performed at baseline (before the first chemotherapy cycle), at the third cycle, and at the sixth cycle, unless otherwise specified.

Primary outcomes (nutritional status) were assessed using two complementary measures:

1. *PG-SGA score*: A validated comprehensive nutrition assessment tool for cancer patients (14). The scale includes two parts: patient self-assessment and medical staff assessment. Scores were interpreted as: 0–1 = no malnutrition, 2–3 = mild malnutrition, 4–8 = moderate malnutrition, and ≥9 = severe malnutrition.
2. *Serum albumin level*: Three mL of fasting venous blood was collected from patients, and serum was separated by centrifugation at 3000 rpm for 10 min. Serum albumin (ALB, g/L) was measured using an automated biochemical analyzer.

Secondary outcomes included a range of anthropometric, hematological, immunological, and patient-reported measures:

1. *Anthropometric measures*: Body mass index (BMI, kg/m^2^) was calculated from measured height and weight. Patients were instructed to record fasting body weight daily using a home body scale and upload records weekly.
2. *Hematological parameters*: Using the same standardized blood samples collected before each chemotherapy cycle (as described above), hemoglobin (Hb, g/L), white blood cell count (×10^9^/L), and platelet count (×10^9^/L) were measured using an automated hematology analyzer (XN-9000, Sysmex, Japan). To account for the potential impact of supportive care on these parameters, we systematically recorded the use of granulocyte colony-stimulating factor (G-CSF), erythropoiesis-stimulating agents (ESA), and blood transfusions throughout the study period for all patients. These data were used to assess whether between-group differences in hematological parameters might be attributable to differential use of supportive care rather than nutritional status alone.
3. *Immune function parameters*: Three mL of fasting venous blood was collected from patients into EDTA-anticoagulated tubes between 7:00 and 8:00 a.m. after an overnight fast, immediately before the start of each chemotherapy cycle (baseline, cycle 3, and cycle 6). This standardized timing was strictly adhered to minimize potential confounding from circadian variations, acute phase responses to recent chemotherapy, and variations in hydration status. The proportions of CD3+, CD4+, and CD8 + T lymphocytes were analyzed by flow cytometry (FACSCanto II, BD Biosciences, USA) within 2 h of collection. Simultaneously, absolute lymphocyte counts were obtained from complete blood count analysis performed on the same samples using an automated hematology analyzer (XN-9000, Sysmex, Japan). Absolute counts of CD4 + and CD8 + T lymphocytes (cells/μL) were then calculated by multiplying the total lymphocyte count by the respective percentage. The CD4+/CD8 + ratio was derived from both percentage and absolute count data. All flow cytometry analyses were performed by trained laboratory technicians who were blinded to group allocation. Quality control procedures were performed daily according to manufacturer specifications.
4. *Cancer-related fatigue*: Assessed using the Piper Fatigue Scale (PFS) (15), which contains 22 items with a total score ranging from 0 to 10. Higher scores indicate more severe fatigue.
5. *Self-care ability*: Evaluated using the Exercise of Self-Care Agency (ESCA) scale (16), which includes four subscales: self-responsibility (0–32 points), nursing skills (0–48 points), self-concept (0–36 points), and health knowledge (0–56 points). The total score ranges from 0 to 172, with higher scores indicating better self-care ability.
6. *Quality of life*: Measured using the General Quality of Life Questionnaire (GQOLI-74) (17), which contains four domains: social function (20–100 points), psychological function (20–100 points), physical function (20–100 points), and material life status (14–60 points). Higher scores represent better quality of life.
7. *Adverse reactions*: Adverse reactions during chemotherapy were recorded and graded according to CTCAE version 5.0 (18), including gastrointestinal reactions, bone marrow suppression, liver function injury, and peripheral nerve injury. Adverse events were identified through daily ward rounds (during hospitalization) and weekly telephone/WeChat follow-up (during home periods) by trained oncology nurses. Laboratory monitoring was performed before each chemotherapy cycle. All adverse event records were independently reviewed by two oncologists blinded to group allocation, with disagreements resolved by consensus. Outcomes included incidence of any adverse reaction, incidence of grade ≥3 reactions, and maximum grade experienced.
8. *Nursing satisfaction*: Assessed at study completion using a self-designed questionnaire. The questionnaire contains 10 items covering four dimensions: nursing attitude (2 items), professional skills (3 items), health guidance (3 items), and response timeliness (2 items). Each item uses a 5-point Likert scale (1 = very dissatisfied to 5 = very satisfied), with total scores ranging from 10–50, converted to a percentage scale (score/50 × 100). Based on percentage scores, satisfaction was categorized as: very satisfied (≥90), satisfied (75–89), general (60–74), and dissatisfied (<60). Nursing satisfaction rate was calculated as: (very satisfied + satisfied cases) / total cases in the group × 100%. The questionnaire demonstrated good internal consistency (Cronbach’s *α* = 0.89).

### Statistical analysis

Analyses were performed using SPSS 26.0 and R 4.1.2 on an intention-to-treat basis, with two-sided *p* < 0.05 considered significant. Baseline characteristics were summarized as mean ± SD or *n* (%).

For primary outcomes (PG-SGA score, serum albumin) and all continuous secondary outcomes (BMI, Hb, WBC, platelets, immune parameters, PFS, ESCA, GQOLI-74), linear mixed-effects models (LMM) with restricted maximum likelihood estimation were employed. LMM included fixed effects of group, time (baseline, cycle 3, cycle 6), group×time interaction, and baseline value as covariate, with patient as random intercept. Unstructured covariance was selected by AIC. Results are presented as estimated marginal means with 95% CIs, *F*-statistics, and *p*-values. Post-hoc pairwise comparisons used Bonferroni correction.

For categorical outcomes (adverse reactions, nursing satisfaction), Pearson’s χ^2^ or Fisher’s exact tests were used; adverse reaction grades were compared by Mann–Whitney U test.

To control type I error for multiple secondary outcomes, Benjamini-Hochberg false discovery rate (FDR) correction was applied (q < 0.05). Effect sizes are reported as mean differences or risk differences with 95% CIs and number needed to treat where appropriate.

Missing data (<2% of assessments) were handled by LMM under missing-at-random assumption. Sensitivity analyses included per-protocol analysis, multiple imputation (MICE with 20 datasets), and adjustment for confounders (tumor site, treatment intent, regimen). Exploratory subgroup analyses examined three-way interactions (group × time × subgroup) for tumor type, treatment intent, and baseline nutritional status.

## Results

### Participant flow and baseline characteristics

Between March 1, 2024, and April 30, 2025, a total of 158 patients with gastrointestinal tumors were assessed for eligibility. Of these, 58 patients were excluded: 32 did not meet inclusion criteria, 20 declined to participate, and 6 were excluded for other reasons (transfer to other hospitals, scheduling conflicts). The remaining 100 eligible patients were randomly assigned to the control group (*n* = 50) or the study group (*n* = 50). All 100 patients received their allocated interventions.

During the 6-month follow-up period, 1 patient in the control group (2.0%) and 2 patients in the study group (4.0%) withdrew consent and were lost to follow-up. These patients completed assessments up to cycle 3 but not cycle 6. Additionally, 8 scheduled assessments (4 in control group, 4 in study group) were missed due to patient scheduling conflicts (<2% of total planned assessments). All 100 patients were included in the intention-to-treat analysis using linear mixed-effects models, which accommodate missing data under the missing-at-random assumption.

### Primary outcomes: nutritional status

**Figure 1:**
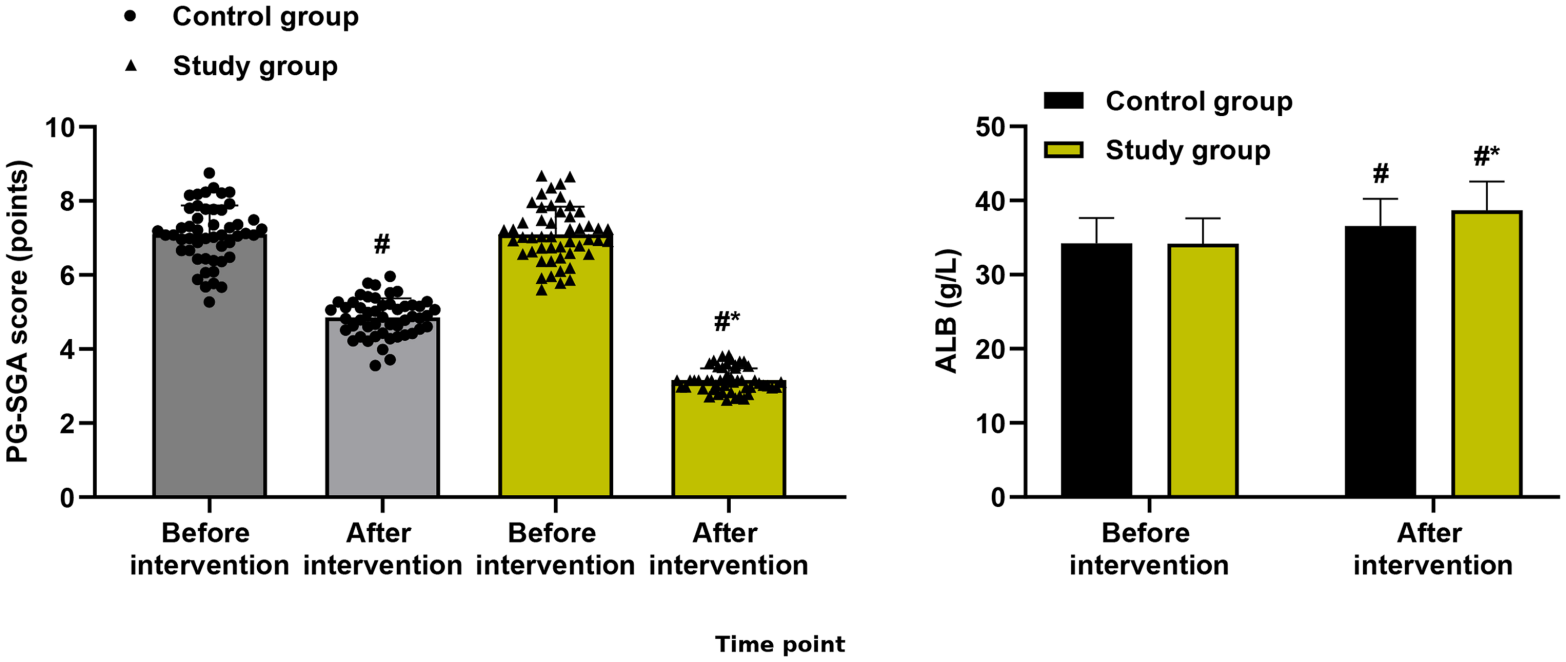
Nutritional status outcomes. PG-SGA score and serum albumin level at baseline, cycle 3, and cycle 6. Data are presented as estimated marginal means with 95% confidence intervals from linear mixed-effects models adjusted for baseline values. *p < 0.05, **p < 0.01, ***p < 0.001 for between-group comparisons at each time point.

For PG-SGA score, linear mixed-effects models revealed a significant group × time interaction (*F* = 14.28, *p* < 0.001). After adjustment for baseline values, the study group demonstrated significantly lower PG-SGA scores at both cycle 3 (estimated marginal mean difference: −1.3, 95% CI: −1.8 to −0.8, *p* < 0.001; Cohen’s d = 0.85) and cycle 6 (estimated marginal mean difference: −1.4, 95% CI: −1.9 to −0.9, *p* < 0.001; Cohen’s d = 0.92), representing large effect sizes (Figure 1).

For serum albumin, a significant group × time interaction was also observed (*F* = 6.92, *p* = 0.002). The study group had significantly higher serum albumin levels at cycle 3 (mean difference: +2.3 g/L, 95% CI: 1.1 to 3.5, *p* < 0.001; Cohen’s d = 0.68) and cycle 6 (mean difference: +2.7 g/L, 95% CI: 1.5 to 3.9, *p* < 0.001; Cohen’s d = 0.82), indicating moderate to large effect sizes (Figure 1).

### Secondary outcomes: anthropometric and hematological parameters

**Figure 2:**
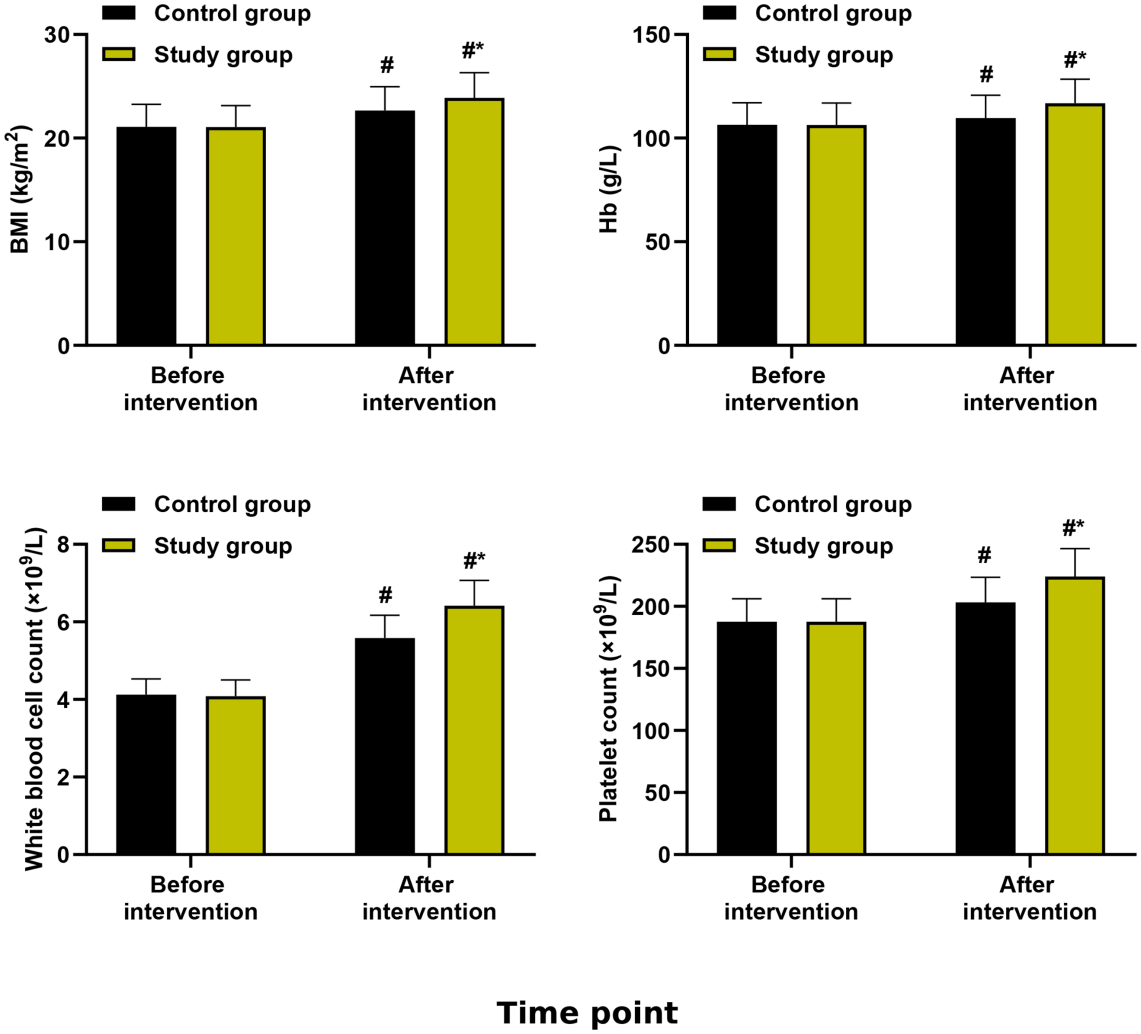
Anthropometric and hematological parameters. Body mass index (BMI), hemoglobin (Hb), white blood cell (WBC) count, and platelet count at baseline, cycle 3, and cycle 6. Data are presented as estimated marginal means with 95% confidence intervals from linear mixed-effects models adjusted for baseline values. *p < 0.05, **p < 0.01 for between-group comparisons at each time point.

For BMI, the group × time interaction was significant (*F* = 4.56, *p* = 0.012). The study group showed higher BMI at cycle 6 compared to the control group (mean difference: +0.8 kg/m^2^, 95% CI: 0.3 to 1.3, *p* = 0.002), but not at cycle 3 (*p* = 0.08) (Table 1; Figure 2).

**Table 1:** Detailed outcomes at each time point for both groups [mean ± SD (95% CI)].

Outcome	Group	*n*	Baseline	*n*	Cycle 3	*n*	Cycle 6	Group × time interaction *F* (*p*)
PG-SGA score	Control	50	5.8 ± 1.5 (5.4–6.2)	49	4.5 ± 1.3 (4.1–4.9)	49	3.8 ± 1.2 (3.4–4.2)	14.28 (<0.001)
	Study	50	5.9 ± 1.6 (5.5–6.3)	48	3.2 ± 1.1 (2.8–3.6)	48	2.5 ± 1.0 (2.1–2.9)	
Serum albumin (g/L)	Control	50	35.2 ± 3.1 (34.3–36.1)	49	37.5 ± 2.8 (36.7–38.3)	49	38.8 ± 2.9 (38.0–39.6)	6.92 (0.002)
	Study	50	35.1 ± 3.2 (34.2–36.0)	48	39.8 ± 3.0 (38.9–40.7)	48	41.5 ± 3.1 (40.6–42.4)	
BMI (kg/m^2^)	Control	50	22.3 ± 2.1 (21.7–22.9)	49	22.5 ± 2.2 (21.9–23.1)	49	22.6 ± 2.3 (22.0–23.2)	4.56 (0.012)
	Study	50	22.4 ± 2.2 (21.8–23.0)	48	23.1 ± 2.3 (22.5–23.7)	48	23.4 ± 2.4 (22.8–24.0)	
Hemoglobin (g/L)	Control	50	118.5 ± 12.3 (115.0–122.0)	49	115.2 ± 13.5 (111.3–119.1)	49	116.8 ± 14.2 (112.7–120.9)	5.23 (0.006)
	Study	50	119.2 ± 13.1 (115.5–122.9)	48	122.5 ± 14.0 (118.4–126.6)	48	125.3 ± 14.5 (121.1–129.5)	
WBC count (×10^9^/L)	Control	50	6.2 ± 1.5 (5.8–6.6)	49	5.4 ± 1.4 (5.0–5.8)	49	5.6 ± 1.5 (5.2–6.0)	4.89 (0.009)
	Study	50	6.3 ± 1.6 (5.9–6.7)	48	6.1 ± 1.5 (5.7–6.5)	48	6.5 ± 1.6 (6.1–6.9)	
Platelet count (×10^9^/L)	Control	50	245 ± 52 (231–259)	49	215 ± 48 (201–229)	49	220 ± 50 (206–234)	4.12 (0.018)
	Study	50	248 ± 54 (233–263)	48	242 ± 51 (227–257)	48	255 ± 53 (240–270)	
CD4 + T cells (cells/μL)	Control	50	620 ± 102 (592–648)	49	590 ± 95 (563–617)	49	580 ± 88 (555–605)	8.45 (<0.001)
	Study	50	615 ± 105 (585–645)	48	645 ± 98 (617–673)	48	680 ± 95 (653–707)	
CD8 + T cells (cells/μL)	Control	50	408 ± 75 (387–429)	49	398 ± 70 (378–418)	49	400 ± 72 (380–420)	1.23 (0.29)
	Study	50	402 ± 78 (380–424)	48	392 ± 73 (371–413)	48	378 ± 65 (359–397)	
CD4+/CD8 + ratio	Control	50	1.52 ± 0.28 (1.44–1.60)	49	1.48 ± 0.25 (1.41–1.55)	49	1.45 ± 0.24 (1.38–1.52)	6.78 (0.002)
	Study	50	1.53 ± 0.29 (1.45–1.61)	48	1.76 ± 0.30 (1.67–1.85)	48	1.80 ± 0.32 (1.71–1.89)	
PFS score	Control	50	5.6 ± 1.8 (5.1–6.1)	49	5.0 ± 1.6 (4.5–5.5)	49	4.5 ± 1.5 (4.1–4.9)	10.23 (<0.001)
	Study	50	5.7 ± 1.9 (5.2–6.2)	48	3.8 ± 1.4 (3.4–4.2)	48	3.0 ± 1.2 (2.7–3.3)	
ESCA total score	Control	50	98.5 ± 12.5 (95.0–102.0)	49	108.2 ± 13.0 (104.5–111.9)	49	115.6 ± 14.2 (111.5–119.7)	9.87 (<0.001)
	Study	50	99.2 ± 13.1 (95.5–102.9)	48	118.5 ± 14.5 (114.3–122.7)	48	131.4 ± 15.8 (126.8–136.0)	
GQOLI-74 total score	Control	50	62.3 ± 8.5 (59.9–64.7)	49	68.5 ± 9.2 (65.9–71.1)	49	72.8 ± 10.1 (69.9–75.7)	8.92 (<0.001)
	Study	50	61.8 ± 8.8 (59.3–64.3)	48	75.2 ± 10.5 (72.2–78.2)	48	82.3 ± 11.5 (79.0–85.6)	

Sample sizes (*n*) vary due to missing data at follow-up time points. All available data were included in linear mixed-effects models. FDR q-values are adjusted for multiple secondary outcomes using the Benjamini-Hochberg procedure. PG-SGA, Patient-Generated Subjective Global Assessment; BMI, Body Mass Index; PFS, Piper Fatigue Scale; ESCA, Exercise of Self-Care Agency; GQOLI-74, General Quality of Life Questionnaire-74.

Significant group × time interactions were observed for hemoglobin (*F* = 5.23, *p* = 0.006), white blood cell count (*F* = 4.89, *p* = 0.009), and platelet count (*F* = 4.12, *p* = 0.018). The study group demonstrated consistently higher values at both post-baseline time points (Table 1). After FDR correction, all these differences remained significant (*q* < 0.05).

To assess whether these differences might be attributable to differential use of supportive care, we compared the utilization of G-CSF, ESA, and blood transfusions between groups. As shown in Table 2, no significant differences were observed between groups in the proportion of patients receiving G-CSF (control: 24.0% vs. study: 20.0%, *p* = 0.63), ESA (control: 6.0% vs. study: 4.0%, *p* = 0.65), or blood transfusions (control: 4.0% vs. study: 2.0%, *p* = 0.56). This suggests that the observed differences in hematological parameters are unlikely to be explained by differential supportive care utilization.

**Table 2:** Baseline characteristics of patients in both groups [x̄ ± s or *n* (%)].

Characteristic	Control group (*n* = 50)	Study group (*n* = 50)	Statistic	*p*-value
Demographics				
Age (years)	49.23 ± 2.45	49.26 ± 2.52	*t* = 0.06	0.95
Sex			χ^2^ = 0.04	0.84
Male	25 (50.0)	24 (48.0)		
Female	25 (50.0)	26 (52.0)		
BMI (kg/m^2^)	22.3 ± 2.1	22.4 ± 2.2	*t* = 0.23	0.82
Tumor characteristics				
Tumor site			χ^2^ = 0.16	0.92
Gastric cancer	23 (46.0)	22 (44.0)		
Colon cancer	15 (30.0)	16 (32.0)		
Rectal cancer	10 (20.0)	10 (20.0)		
Other*	2 (4.0)	2 (4.0)		
Tumor stage (AJCC 8th)			χ^2^ = 0.04	0.98
Stage II	15 (30.0)	14 (28.0)		
Stage III	32 (64.0)	33 (66.0)		
Stage IV	3 (6.0)	3 (6.0)		
Histological grade			χ^2^ = 0.17	0.92
Well/moderately differentiated	28 (56.0)	27 (54.0)		
Poorly differentiated	22 (44.0)	23 (46.0)		
Treatment characteristics				
Treatment intent			χ^2^ = 0.16	0.69
Neoadjuvant chemotherapy	28 (56.0)	26 (52.0)		
Adjuvant chemotherapy	22 (44.0)	24 (48.0)		
Chemotherapy regimen				
SOX	12 (24.0)	11 (22.0)	χ^2^ = 0.06	0.81
XELOX	23 (46.0)	25 (50.0)	χ^2^ = 0.16	0.69
FOLFOX6	10 (20.0)	9 (18.0)	χ^2^ = 0.07	0.80
FOLFIRI	5 (10.0)	5 (10.0)	χ^2^ = 0.00	1.00
Planned chemotherapy cycles	6.2 ± 1.1	6.1 ± 1.2	*t* = 0.44	0.66
Baseline nutritional status				
PG-SGA score	5.8 ± 1.5	5.9 ± 1.6	*t* = 0.32	0.75
PG-SGA category			χ^2^ = 0.04	0.98
Mild malnutrition (2–3)	28 (56.0)	27 (54.0)		
Moderate malnutrition (4–8)	22 (44.0)	23 (46.0)		
Serum albumin (g/L)	35.2 ± 3.1	35.1 ± 3.2	*t* = 0.16	0.87
Hemoglobin (g/L)	118.5 ± 12.3	119.2 ± 13.1	*t* = 0.28	0.78
Comorbidities				
Hypertension	8 (16.0)	7 (14.0)	χ^2^ = 0.08	0.78
Coronary heart disease	3 (6.0)	4 (8.0)	χ^2^ = 0.15	0.70
Chronic liver disease	2 (4.0)	1 (2.0)	χ^2^ = 0.34	0.56
Chronic kidney disease	1 (2.0)	1 (2.0)	χ^2^ = 0.00	1.00
Supportive care during study				
G-CSF use	12 (24.0)	10 (20.0)	χ^2^ = 0.23	0.63
ESA use	3 (6.0)	2 (4.0)	χ^2^ = 0.21	0.65
Blood transfusion	2 (4.0)	1 (2.0)	χ^2^ = 0.34	0.56
Corticosteroid use (antiemetic)	50 (100.0)	50 (100.0)	—	—

“Other” includes esophageal cancer (*n* = 2) and gastrointestinal stromal tumor (*n* = 2). AJCC, American Joint Committee on Cancer; PG-SGA, Patient-Generated Subjective Global Assessment; G-CSF, Granulocyte colony-stimulating factor; ESA, Erythropoiesis-stimulating agent.

### Secondary outcomes: immune function

Linear mixed-effects models revealed significant group × time interactions for CD4 + T lymphocyte absolute count (*F* = 8.45, *p* < 0.001) and CD4+/CD8 + ratio (*F* = 6.78, *p* = 0.002), but not for CD8 + T lymphocyte absolute count (*F* = 1.23, *p* = 0.29). These findings remained significant after FDR correction for multiple secondary outcomes (*q* < 0.05).

**Figure 3:**
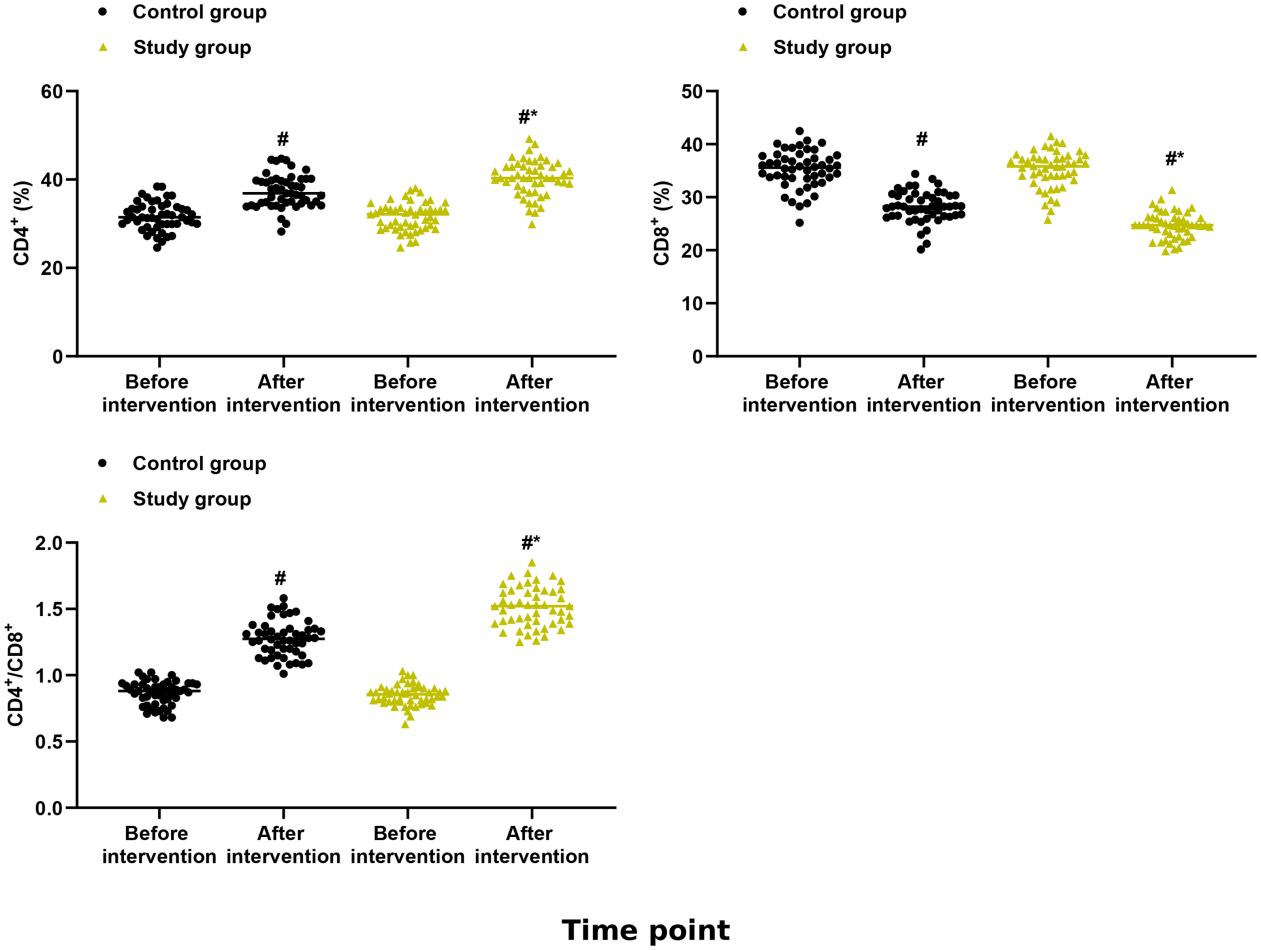
Immune function outcomes. CD4^+^ T lymphocyte absolute count and CD4^+^/CD8^+^ ratio at baseline, cycle 3, and cycle 6. Data are presented as estimated marginal means with 95% confidence intervals from linear mixed-effects models adjusted for baseline values. *p < 0.05, **p < 0.01, ***p < 0.001 for between-group comparisons at each time point.

At baseline, no significant differences were observed between groups in any immune parameters (all *p* > 0.05). After intervention, the study group demonstrated significantly higher CD4 + T lymphocyte absolute counts at cycle 6 compared to the control group (study group: 680 ± 95 cells/μL [95% CI, 653 to 707]; control group: 580 ± 88 cells/μL [95% CI, 555 to 605]; mean difference: +95 cells/μL, 95% CI: 45 to 145, *p* < 0.001) (Figure 3). The CD4+/CD8 + ratio was significantly higher in the study group at both cycle 3 (study group: 1.76 ± 0.30 [95% CI, 1.67 to 1.85]; control group: 1.48 ± 0.25 [95% CI, 1.41 to 1.55]; mean difference: +0.28, 95% CI: 0.12 to 0.44, *p* < 0.001) and cycle 6 (study group: 1.80 ± 0.32 [95% CI, 1.71 to 1.89]; control group: 1.45 ± 0.24 [95% CI, 1.38 to 1.52]; mean difference: +0.35, 95% CI: 0.19 to 0.51, *p* < 0.001) (Figure 3).

CD8 + T lymphocyte absolute counts did not differ significantly between groups at any time point (cycle 6: study group 378 ± 65 cells/μL vs. control group 400 ± 72 cells/μL, *p* = 0.12). The observed changes in CD4+/CD8 + ratio were therefore driven primarily by increases in CD4 + T cells rather than decreases in CD8 + T cells.

### Secondary outcomes: cancer-related fatigue, self-care ability, and quality of life

**Figure 4:**
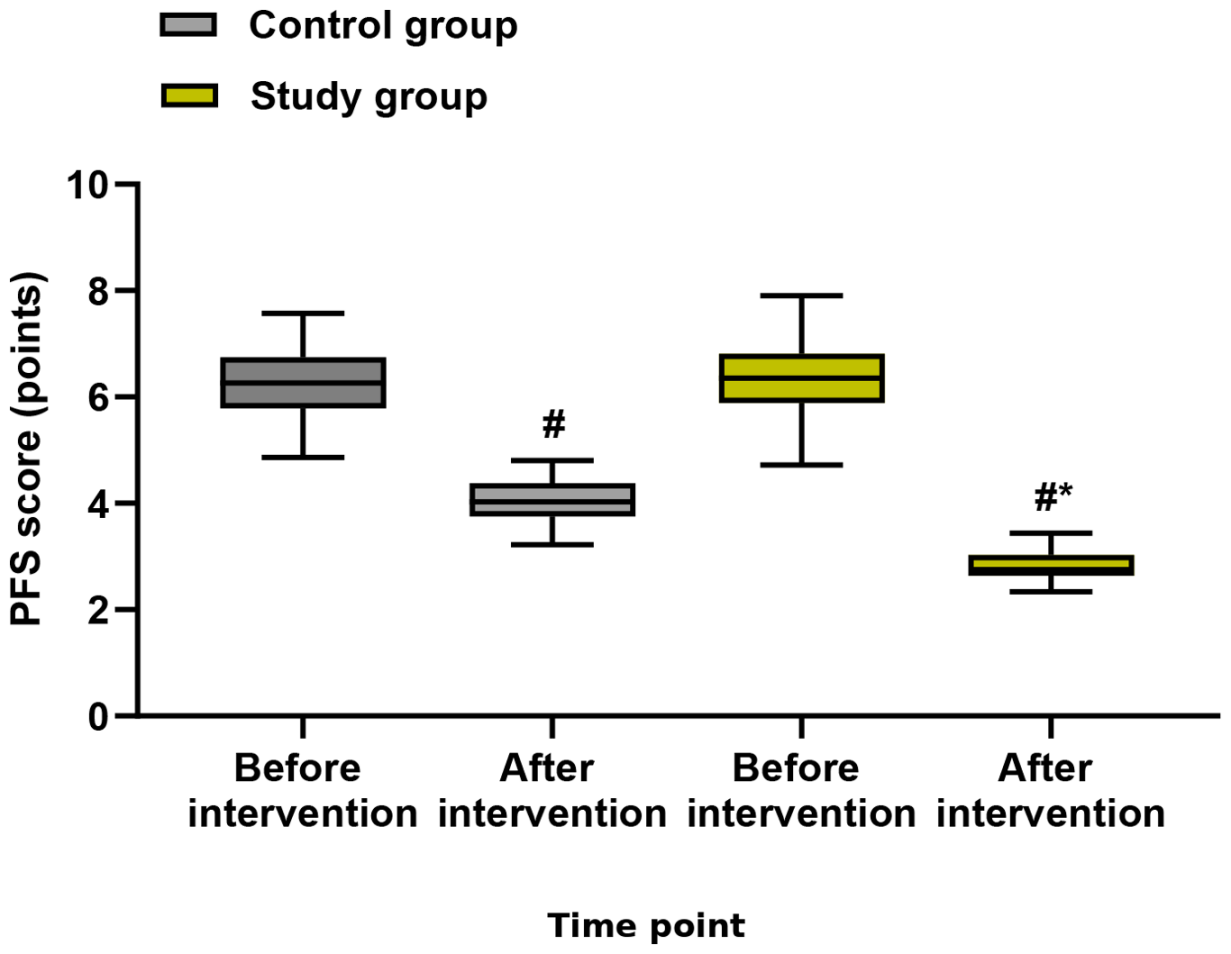
Cancer-related fatigue (PFS score) at baseline, cycle 3, and cycle 6. Data are presented as estimated marginal means with 95% confidence intervals from linear mixed-effects models adjusted for baseline values. ***p < 0.001 for between-group comparisons at each time point.

For PFS score (cancer-related fatigue), the group × time interaction was significant (*F* = 10.23, *p* < 0.001). The study group demonstrated significantly lower fatigue scores at both cycle 3 (mean difference: −1.2, 95% CI: −1.7 to −0.7, *p* < 0.001) and cycle 6 (mean difference: −1.5, 95% CI: −2.0 to −1.0, *p* < 0.001) (Figure 4).

**Figure 5:**
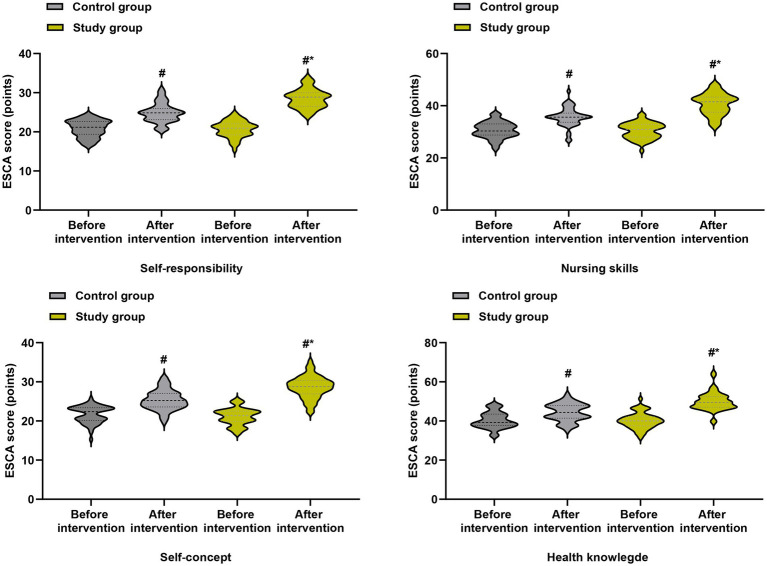
Self-care ability (ESCA total score) at baseline, cycle 3, and cycle 6. Data are presented as estimated marginal means with 95% confidence intervals from linear mixed-effects models adjusted for baseline values. ***p < 0.001 for between-group comparisons at each time point.

**Figure 6:**
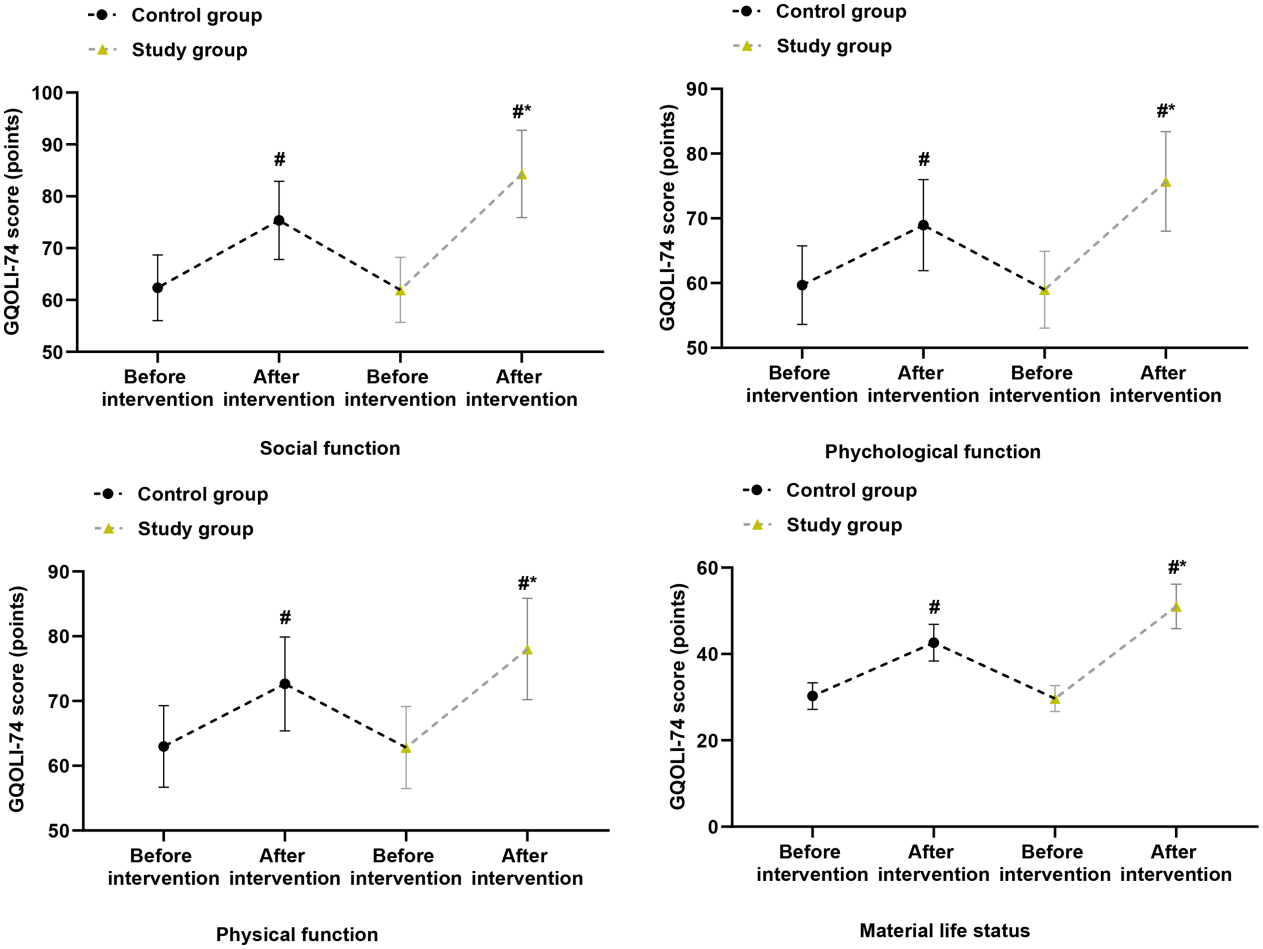
Quality of life (GQOLI-74 total score) at baseline, cycle 3, and cycle 6. Data are presented as estimated marginal means with 95% confidence intervals from linear mixed-effects models adjusted for baseline values. ***p < 0.001 for between-group comparisons at each time point.

For ESCA total score and all subscales (self-responsibility, nursing skills, self-concept, health knowledge), significant group × time interactions were observed (all *p* < 0.01), with the study group showing higher scores at both post-baseline time points (Figure 5). Similarly, for GQOLI-74 total score and all subscales (social function, psychological function, physical function, material life status), significant group × time interactions were found (all *p* < 0.01), favoring the study group (Figure 6). All these differences remained significant after FDR correction (*q* < 0.05).

### Secondary outcomes: adverse reactions

The overall incidence of adverse reactions was significantly lower in the study group compared to the control group (4.0% vs. 20.0%, χ^2^ = 6.06, *p* = 0.01; risk difference: −16.0, 95% CI: −28.5% to −3.5%; Table 3). No grade 3–4 adverse events were observed in the study group, while the control group experienced three grade 3 events (two neutropenia, one nausea/vomiting). The maximum grade of adverse events was significantly lower in the study group (Mann–Whitney U test: *p* = 0.02). Individual adverse reaction categories showed consistent directional effects favoring the study group, although differences did not reach statistical significance due to low event rates: gastrointestinal reactions (2.0% vs. 6.0%, *p* = 0.31), bone marrow suppression (2.0% vs. 4.0%, *p* = 0.56), liver function injury (0% vs. 6.0%, *p* = 0.08), and peripheral nerve injury (0% vs. 4.0%, *p* = 0.15). No serious adverse events occurred in either group.

**Table 3:** Adverse reactions in both groups [*n* (%)] with risk differences and 95% CIs.

Adverse reaction category	Control group (*n* = 50)	Study group (*n* = 50)	Risk difference (95% CI)	χ^2^	*p*-value
Any adverse reaction	10 (20.0)	2 (4.0)	-16.0% (−28.5% to −3.5%)	6.06	0.01
Grade ≥3 adverse reactions	3 (6.0)	0 (0)	−6.0% (−12.6 to 0.6%)*	3.09	0.08*
Gastrointestinal reactions	3 (6.0)	1 (2.0)	−4.0% (−11.6 to 3.6%)	1.04	0.31
Nausea/vomiting	2 (4.0)	1 (2.0)	−2.0% (−8.6 to 4.6%)	0.34	0.56
Diarrhea	1 (2.0)	0 (0)	−2.0% (−5.9 to 1.9%)*	1.01	0.32*
Bone marrow suppression	2 (4.0)	1 (2.0)	−2.0% (−8.6 to 4.6%)	0.34	0.56
Anemia	1 (2.0)	1 (2.0)	0% (−5.5 to 5.5%)	0.00	1.00
Neutropenia	2 (4.0)	0 (0)	−4.0% (−9.4 to 1.4%)*	2.04	0.15*
Thrombocytopenia	0 (0)	0 (0)	-	-	-
Liver function injury	3 (6.0)	0 (0)	−6.0% (−12.6 to 0.6%)*	3.09	0.08*
Peripheral nerve injury	2 (4.0)	0 (0)	−4.0% (−9.4 to 1.4%)*	2.04	0.15*
Maximum grade experienced				Z = −2.32^†^	0.02
Grade 0	40 (80.0)	48 (96.0)	+16.0% (3.5 to 28.5%)		
Grade 1–2	7 (14.0)	2 (4.0)	−10.0% (−21.2 to 1.2%)		
Grade 3–4	3 (6.0)	0 (0)	−6.0% (−12.6 to 0.6%)		

Fisher’s exact test used; ^†^Mann–Whitney U test; CI, confidence interval.

### Secondary outcomes: nursing satisfaction

The nursing satisfaction rate was significantly higher in the study group compared to the control group (96.0% vs. 80.0%, χ^2^ = 6.06, *p* = 0.01; risk difference: +16.0%, 95% CI: 3.5 to 28.5%; Table 4). The distribution of satisfaction categories differed significantly between groups (Mann–Whitney U test: *p* = 0.01), with the study group having more very satisfied patients (46.0% vs. 40.0%) and fewer dissatisfied patients (0% vs. 4.0%). Dimension-specific analysis revealed significantly higher scores in the study group across all four dimensions: nursing attitude (4.6 ± 0.4 vs. 4.2 ± 0.6, *p* < 0.001), professional skills (4.5 ± 0.5 vs. 4.1 ± 0.7, *p* < 0.001), health guidance (4.7 ± 0.4 vs. 4.0 ± 0.8, *p* < 0.001), and response timeliness (4.5 ± 0.5 vs. 4.0 ± 0.7, *p* < 0.001).

**Table 4:** Nursing satisfaction in both groups.

Satisfaction measure	Control group (*n* = 50)	Study group (*n* = 50)	Difference (95% CI)	Statistic	*p*-value
Satisfaction category [*n* (%)]				Z = −2.48*	0.01
Very satisfied	20 (40.0)	23 (46.0)	+6.0% (−13.5 to 25.5%)		
Satisfied	20 (40.0)	25 (50.0)	+10.0% (−9.5 to 29.5%)		
General	8 (16.0)	2 (4.0)	−12.0% (−23.8% to −0.2%)		
Dissatisfied	2 (4.0)	0 (0)	−4.0% (−9.4 to 1.4%)^†^		
Nursing satisfaction rate [*n* (%)]	40 (80.0)	48 (96.0)	+16.0% (3.5 to 28.5%)	χ^2^ = 6.06	0.01
Dimension-specific scores (mean ± SD)					
Nursing attitude (2–10)	8.4 ± 1.2	9.2 ± 0.8	+0.8 (0.4 to 1.2)	*t* = 3.92	<0.001
Professional skills (3–15)	12.3 ± 2.1	13.5 ± 1.5	+1.2 (0.5 to 1.9)	*t* = 3.28	0.001
Health guidance (3–15)	12.0 ± 2.4	14.1 ± 1.2	+2.1 (1.3 to 2.9)	*t* = 5.46	<0.001
Response timeliness (2–10)	8.0 ± 1.4	9.0 ± 1.0	+1.0 (0.5 to 1.5)	*t* = 4.08	<0.001
Total raw score (10–50)	40.7 ± 6.2	45.8 ± 3.5	+5.1 (3.1 to 7.1)	*t* = 5.02	<0.001
Percentage score (%)	81.4 ± 12.4	91.6 ± 7.0	+10.2 (6.2 to 14.2)	*t* = 5.02	<0.001

Mann–Whitney U test; ^†^Fisher’s exact test; CI, confidence interval.

### Sensitivity analyses

Sensitivity analyses using per-protocol analysis (*n* = 92 after excluding 8 patients with major protocol violations) and multiple imputation for missing data yielded results consistent with the primary intention-to-treat analysis (all interaction *p*-values remained <0.05 for primary outcomes). Adjustment for tumor site, treatment intent, and chemotherapy regimen as covariates in the LMM did not materially change the effect estimates (change in coefficient <10% for all outcomes), supporting the robustness of our findings.

### Subgroup analyses

Exploratory subgroup analyses suggested that the intervention effect on PG-SGA score change was consistent across tumor types (gastric vs. colorectal: three-way interaction *p* = 0.42), treatment intent (neoadjuvant vs. adjuvant: *p* = 0.38), and baseline nutritional status (PG-SGA 2–3 vs. 4–8: *p* = 0.51), indicating that the H2H nutrition management model may be broadly effective across these subgroups.

## Discussion

Chemotherapy is cyclical, with patients spending 2–3 weeks at home between cycles to recover from adverse effects (19). This home period represents an optimal window for nutritional intervention (20). However, discharge often interrupts professional nutrition support, potentially exacerbating malnutrition (21). Therefore, extending nutrition management from hospital to home through standardized, whole-process approaches has become a critical challenge.

The H2H nutrition management model provides continuous, individualized care from hospital to home, emphasizing patient and caregiver engagement (22). It follows a structured process of screening, assessment, intervention, and monitoring. Originating from the American Heart College’s H2H study (23), this model is primarily implemented by nutrition support teams comprising clinicians, nutritionists, and nurses in international settings (24). Researchers have successfully applied H2H to patients undergoing cancer surgery, chemotherapy, and radiotherapy (25). Qiu et al. (7) demonstrated that H2H reduces complications, improves treatment completion, and extends professional care beyond hospitalization. Accordingly, we evaluated its effects on nutritional status and immune function in gastrointestinal cancer patients receiving chemotherapy.

A key strength of this study is its rigorous statistical methodology, addressing common limitations in nutrition research. Unlike simple pre-post *t*-tests, we employed linear mixed-effects models for all repeated outcomes, offering multiple advantages: (1) accounting for within-subject correlation; (2) handling missing data under missing-at-random assumption; (3) adjusting for baseline values; and (4) testing group × time interactions. Significant interactions for primary outcomes (PG-SGA: *F* = 14.28, *p* < 0.001; albumin: *F* = 6.92, *p* = 0.002) provide robust evidence of sustained H2H benefits over 6 months. Effect sizes at cycle 6 (PG-SGA: −1.4 points, 95% CI: −1.9 to −0.9; albumin: +2.7 g/L, 95% CI: 1.5 to 3.9) were clinically meaningful and comparable to previous studies (26). We addressed multiple comparisons by pre-specifying co-primary endpoints and applying Benjamini-Hochberg FDR correction; all significant secondary outcomes remained significant after adjustment (q < 0.05). Sensitivity analyses (per-protocol, multiple imputation, confounder adjustment) yielded consistent results, supporting robustness. Exploratory subgroup analyses suggested consistent effects across tumor types (*p* = 0.42), treatment intent (*p* = 0.38), and baseline nutritional status (*p* = 0.51), indicating broad applicability, though these findings require cautious interpretation due to limited sample size.

Improvements in hemoglobin, white blood cells, and platelets in the study group warrant cautious interpretation. While these parameters often reflect nutritional status, they are directly influenced by chemotherapy-induced myelosuppression and supportive care (G-CSF, ESA, transfusions). Supportive care use was balanced between groups, making differential treatment an unlikely explanation. However, limitations remain: (1) we did not adjust for timing, dosage, or duration of these interventions; (2) subclinical infections or inflammation were not assessed; (3) iron, B12, and folate status were not measured; (4) platelet improvements, though significant, were modest with uncertain clinical significance (no bleeding events). Thus, these findings are hypothesis-generating, suggesting nutritional support may enhance hematopoietic function, but requiring confirmation with comprehensive bone marrow assessments.

The H2H model significantly improved immune function, with higher CD4 + counts and CD4+/CD8 + ratios in the study group. CD4 + T cells are crucial for antitumor immunity and infection defense; their depletion predicts poorer outcomes in chemotherapy patients. Interpretation requires consideration of confounding factors: (1) lymphocyte subsets are influenced by hydration, corticosteroids, infections, and sampling timing—we minimized these by standardized fasting morning collections, reporting absolute counts, and documenting corticosteroid use; (2) all patients received dexamethasone, which induces transient lymphopenia; uniform exposure suggests between-group differences reflect true intervention effects, though absolute CD4 + counts may be attenuated; (3) the +95 cells/μL increase (16% relative) at cycle 6, while modest, may be clinically relevant—previous studies associate even modest CD4 + improvements with reduced infections and better treatment tolerance (27); (4) nutrition-immunity relationships are bidirectional and complex; our findings align with this but cannot establish causality; (5) functional immune parameters (proliferation, cytokine production) were not assessed. Future studies should incorporate functional assays and longer follow-up to determine if immunological improvements translate to reduced infections or better survival.

Our study also demonstrated significant benefits in patient-reported outcomes. PFS scores (cancer-related fatigue) decreased more in the study group, while ESCA (self-care) and GQOLI-74 (quality of life) scores increased significantly, consistent with Blanco et al. (28). These findings suggest H2H nutrition management effectively reduces fatigue and enhances patients’ capacity for self-management and overall well-being during chemotherapy.

Furthermore, the study group showed lower adverse reaction rates (4.0% vs. 20.0%, *p* = 0.01) and higher nursing satisfaction (96.0% vs. 80.0%, *p* = 0.01), aligning with Oksholm et al. and Bohra et al. (29, 30). Reduced toxicity may reflect improved nutritional status enhancing physiological resilience, while higher satisfaction likely stems from continuous, personalized support and comprehensive health guidance provided by the H2H model.

### Limitations

Several limitations should be acknowledged. First, blinding was not feasible due to intervention nature, potentially introducing performance and detection bias for subjective outcomes despite mitigation strategies. Second, despite enrolling homogeneous gastric/colorectal cancer populations, heterogeneity persists in treatment settings and regimens, though balanced between groups and addressed by mixed models. Third, small sample size (*n* = 100) precluded subgroup analyses by tumor type or regimen, limiting identification of optimal candidates for H2H intervention. Fourth, supportive care medications were balanced but not adjusted in primary analyses due to limited events, leaving residual confounding possible. Fifth, immune assessment was limited to lymphocyte counts without functional assays (proliferation, cytokine production), warranting future comprehensive evaluation. Sixth, residual confounding from infections, inflammation, or steroid metabolism cannot be excluded despite standardized sampling. Seventh, missing data was minimal (<2%) and handled by mixed models and multiple imputation, with consistent results mitigating bias concerns. Eighth, gastric and colorectal cancer patients may have different nutritional challenges, though subgroup analysis showed consistent effects (*p* = 0.42) but was underpowered. Ninth, exclusion of liver/pancreatic cancer limits generalizability; these patients have distinct nutritional issues requiring separate evaluation. Tenth, lack of long-term oncological outcomes (survival, recurrence) prevents determining if nutritional improvements translate to better prognosis; 3–5 year follow-up needed.

## Conclusion

In conclusion, this randomized controlled trial demonstrates that whole-cycle nutrition management based on the “H2H” model significantly improves nutritional status and patient-reported outcomes in patients undergoing chemotherapy for gastrointestinal tumors. The intervention was associated with modest improvements in CD4 + T lymphocyte counts and hematological parameters, suggesting potential benefits for immune function and hematopoietic recovery, although these findings should be interpreted cautiously given the multiple factors that influence these parameters. The H2H model represents a promising approach to bridging the gap between hospital-based and home-based care for cancer patients receiving cyclical chemotherapy. Future large-scale, multicenter studies with larger sample sizes (e.g., 200–300 patients per group), longer follow-up (including 3–5 year survival outcomes), and more comprehensive immune function assessments are warranted to confirm these findings, to establish the long-term prognostic significance of the H2H model, and to identify which patient subgroups may derive the greatest benefit from this intervention.

## Data Availability

The original contributions presented in the study are included in the article/Supplementary material, further inquiries can be directed to the corresponding author.

## Supplementary material

SUPPLEMENTARY FIGURE S1 Participant flow diagram. A total of 158 patients were assessed for eligibility, 100 were randomized, and all were included in the intention-to-treat analysis. Loss to follow-up: control group *n* = 1, study group *n* = 2.
