# Supplementary figures and images for: Effects of whole cycle nutrition management based on “Hospital to Home (H2H)” model on nutritional status and immune function of patients with gastrointestinal tumor chemotherapy
Source: Front Med (Lausanne). 2026 Mar 31;13:1775700. doi: 10.3389/fmed.2026.1775700 (PMC13076122; doi:10.3389/fmed.2026.1775700)

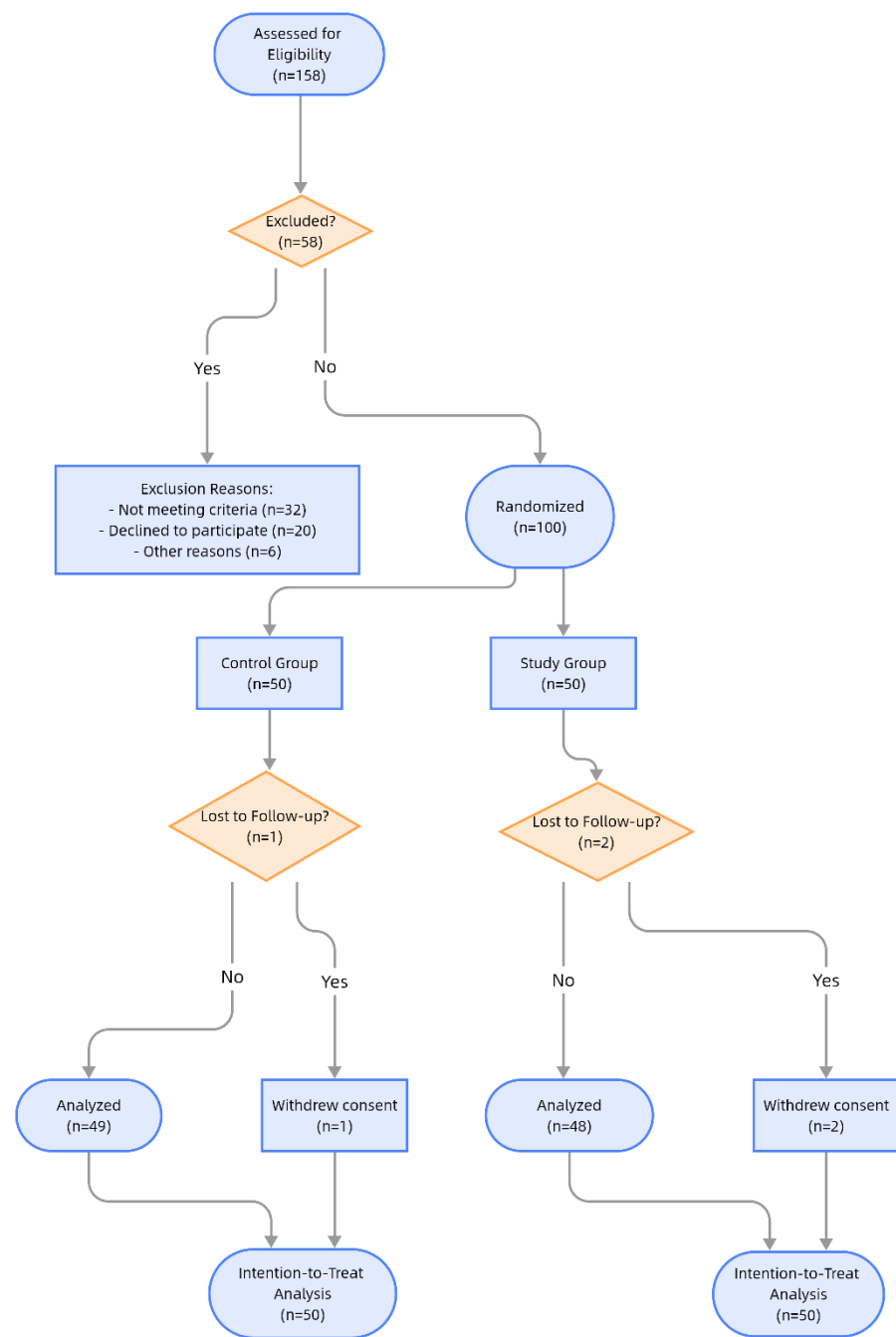

Supplement: SUPPLEMENTARY FIGURE S1 — Participant flow diagram. A total of 158 patients were assessed for eligibility, 100 were randomized, and all were included in the intention-to-treat analysis. Loss to follow-up: control group n = 1, study group n = 2. [file Data_Sheet_1.pdf]
